# The Role of Point-of-Care Testing to Improve Acute Care and Health Care Services

**DOI:** 10.7759/cureus.55315

**Published:** 2024-03-01

**Authors:** Islam H Elrobaa, Keebat Khan, Eslam Mohamed

**Affiliations:** 1 Emergency Medicine, College of Medicine, Qatar University, Doha, QAT; 2 Emergency Medicine, Hamad Medical Corporation (HMC), Doha, QAT

**Keywords:** patient triage, early deposition, early management, early diagnosis, overcrowded, waiting time, short stay, turnaround time (tat), point of care testing

## Abstract

Health care is one of the most important services that need to be provided to any community. Many challenges exist in delivering proper and effective health services, including ensuring timely delivery, providing adequate care through effective management and achieving good outcomes. Point-of-care testing (POCT) plays a crucial role in delivering urgent and appropriate health services, especially in peripheral communities, emergency situations, disaster areas and overcrowded areas. We collected and reviewed secondary data about point-of-care testing from PubMed, Scopus and Google Scholar. Our findings emphasize that POCT provides fast care with minimal waiting time, avoids unnecessary investigations, aids in triage, and provides decision-makers with a clear understanding of the patient’s condition to make informed decisions. We recommend point-of-care testing as a frontline investigation in emergency departments, intensive care units, peripheral hospitals, primary health care centers, disaster areas and field hospitals. Point-of-care testing can improve the quality of health services and ensure the provision of necessary health care.

## Introduction and background

Point-of-care (POC) testing (POCT) refers to simple or non-invasive diagnostic tests that are conducted near the patients, eliminating the need to send samples to a central lab or tertiary hospital for analysis. The point-of-care procedure is characterized by a swift turnaround time, often yielding results within a few minutes. Any simple and non-invasive diagnostic test or procedure that is administered near the patient can be classified as point-of-care [1,2]. 

POCT can be used in emergency departments (EDs), intensive care units (ICUs), acute care services, primary health care, pre-hospital settings, rural hospitals, mobile medical units, field hospitals during disasters, military camps, and overcrowded or mass-gathering areas. It is considered the front line for triage and patient management. Point-of-care testing requires trained staff, such as doctors, staff nurses, nursing aides, and paramedics, who can use the point-of-care procedures. Also, it needs flexible equipment. Point-of-care testing includes venous blood gas (VBG), random blood sugar (RBS), POC pregnancy test, POC urine analysis, POC troponin, POC D-dimer, and POC machines, such as portable X-ray, ECG, pulse oximetry, and portable ultrasound [3-5].

POCT can improve the quality of healthcare services in the ED by reducing the duration of stay. Prolonged stays in the EDs often stem from factors such as overcrowding, waiting for investigations and results, time needed for pain management, waiting for consultations, waiting for admissions or bed availability, and, in rare cases, unclear plans or diagnoses. The point-of-care unit can provide rapid results and give a general idea about the patient’s condition within minutes. This leads to a clearer treatment plan, enables more patients to be examined by emergency physicians, assists decision-makers during triage to prioritize cases, and expedites the consultation or transfer process [6,7].

POCT is helpful in ICUs, resuscitation rooms and high acuity units by providing rapid insights into critical or deteriorating cases. This aids in formulating timely and appropriate treatment plans. Key POCT parameters include blood gases, troponin T, D-dimer, C-reactive protein (CRP), pro-brain natriuretic peptide (proBNP), creatinine, and ultrasound or echocardiography, which are particularly helpful in understanding patient conditions to make early diagnosis and initiate stabilization [8].

Peripheral hospitals in remote areas typically need cost-effective and efficient equipment to diagnose simple, uncomplicated and severe cases. They also triage patients who need to be transferred to a tertiary hospital or can be treated locally. Therefore, they can use POCT to diagnose and treat uncomplicated cases, as well as stabilize and transfer critical cases [9].

During disasters, in camps, field hospitals, and military hospitals, POCT is helpful to diagnose and manage the causalities. It offers flexibility in transportation, allowing it to be deployed anywhere, with minimal waiting time for consistently accurate results [10].

When using POCT, some factors should be considered to ensure high-quality results and minimize medical errors. The operator conducting the POCT should undergo extensive training. Clinical judgement supersedes POCT results, as false positives or negatives should be considered. Clinicians may use multiple POC diagnostic tests to establish an accurate diagnosis and determine whether the patient needs further investigations are necessary or can be managed locally. Additionally, proper documentation should be emphasized when using POCT [11].

In this article, we reviewed POCT types, uses, benefits, and how to ensure high-quality results when used in healthcare services.

## Review

Materials and methods

This is a narrative review focused on POCT. It is a descriptive study to mention the story of POCT. We collected a qualitative data about POCT. We used secondary data collected from articles about POCT, including original studies and reviews, indexed in PubMed, Scopus and Google Scholar. Our search specifically targeted POCT, point-of-care ultrasound (POCUS), and POCT uses in the ED. Our research question centered on the impact of POCT on healthcare and acute care services, examining its role in improvement. The primary outcome sought to understand how POCT improves healthcare and acute care services. The secondary outcomes explored the uses of the POCT in clinical management. The final outcome explored strategies to prevent clinical errors by ensuring high-quality results with POCT. We analyzed our data into six sections: what is POCT, what are the advantages of POCT, where can we use POCT, how we can use POCT in clinical management, what are the disadvantages of POCT, and how can we ensure high-quality POCT results without clinical errors.

Results

We analyzed our data into six sections: 1- the definition and types of POCT, 2- the advantages of point of care testing, 3- the uses of point of care testing, 4 -clinical management with POCT, 5- the disadvantages of POCT, 6- how to get high-quality POCT results without clinical errors.

The Definition and Types of POCT

**Figure 1:**
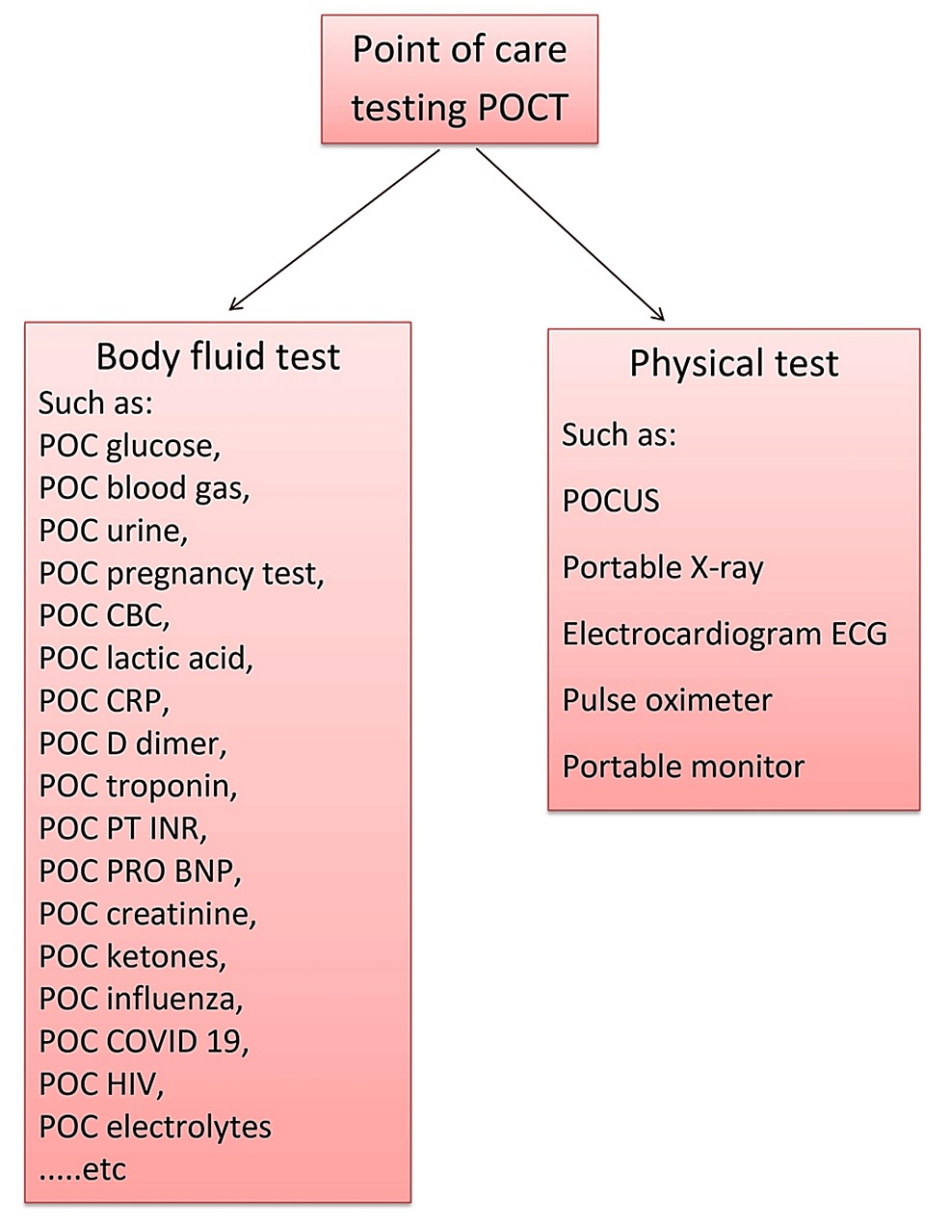
Types of POCT POCT: point-of-care testing, CBC: complete blood count, CRP: C-reactive protein, PT INR: prothrombin time - international normalised ratio, proBNP: pro-brain natriuretic peptide, POCUS: point-of-care ultrasound

Point-of-care means near the patient. It refers to any diagnostic test administered near the patient, eliminating the need to send samples to a central laboratory. We can categorize POCT into two categories: physical POCT and body fluid POCT, encompassing urine, blood, or nasopharyngeal specimens. Physical POCT includes devices such as pulse oximeters, blood pressure machines, electrocardiogram machines, and portable monitors. The portable X-ray machine can also be considered as a physical POCT. Body fluid POCT includes blood tests, such as RBG, venous or arterial blood gases, POC troponin T or I, POC coagulation (international normalised ratio (INR) and partial thromboplastin time (PTT)), POC D-dimer, POC proBNP, POC creatinine, POC blood count, and POC ketones. It also involves POC urine and pregnancy tests. The authors highly value the POC antigen test for coronavirus disease 2019 (COVID-19) and influenza viruses, providing rapid results for patients with respiratory infections and fever. POCUS is considered a physical POCT, playing a crucial role in diagnosing various acute diseases (Table 1). Some authors regard the pocket-size ultrasound as the future stethoscope (Figure 1) [1,2,12].

**Table 1:** The Benefits of Point-of-Care Testing (POCT) *Turnaround time is the time or period between the sample collection from the patient and the result becoming ready to inform the patient or the health care provider, so it’s from the patient to the test operator, then to the patient again. Almost all POCT turnaround times are less than 30 minutes.

The Point of Care Test	The Benefits	*Turnaround Time	Where To Use It	When To Use It
POC venous/atrial blood gases (VBG/ABG)	To detect pH level. To rule out electrolyte imbalance (sodium and potassium). To rule out hypoxia or respiratory failure type 1. To check the carbon dioxide (CO2) level. To check bicarbonate level. To check lactic acid level.	About 15 minutes [43]	Pre-hospital, hospital and anywhere.	In any acute case, to evaluate and monitor the chronic or acute case of respirator, cardiac, gastroenterology, urology, nephrology, any surgical or medical case.
POC urine test	To rule urinary tract infection. To rule out haematuria. To check urine nitrate. To check urine protein. To check urine red blood cells RBCs. To check urine white blood cells WBCs. To check urine glucose. To check urine ketones.	Within minutes after sample collection	Pre-hospital, hospital and anywhere.	In any acute case, renal and fever case.
POC urine pregnancy test/urine hCG test	To rule out pregnancy.	Takes a few minutes	Pre-hospital, hospital and anywhere.	In any woman who suspects pregnancy.
POC pro-brain natriuretic peptide (proBNP)	To rule out heart failure.	About 12-20 minutes [44]	Pre-hospital, hospital and anywhere.	In any acute case, as needed in lower respiratory illness, volume overload and cardiac cases.
POC troponin	To rule out myocardial infraction.	Within 20 minutes [45]	Pre-hospital, hospital and anywhere.	In acute cases, in any chest pain, abdominal pain and back of chest pain. In neurovascular syndrome suspicion cases.
POC D dimer	To rule out deep venous thrombosis. To rule out pulmonary embolism. To rule out aortic dissection. To rule out thromboembolic manifestations.	Less than 21 minutes [46]	Pre-hospital, hospital and anywhere.	In any acute critical case, shortness of breath, chest pain, DVT suspicion, pulmonary embolism suspicion, aortic dissection suspicion.
POC glucose	To rule out hypo- or hyperglycaemia.	May be less than one minute	Pre-hospital, hospital and anywhere.	In any loss of consciousness or disturbed conscious level cases, in diabetic cases, in any critical illness case.
POC ketones	To rule out ketosis.	Within a few minutes	Pre-hospital, hospital and anywhere.	In hyperglycaemic cases, acute abdominal pain and vomiting.
POC creatinine	To rule out renal failure. To rule out renal impairment. To rule out acute kidney injury.	About 9 minutes or less [47]	Pre-hospital, hospital and anywhere.	In any acute case, abdominal pain, vomiting, muscular pain, as needed in any case
POC lactate	To rule out lactic acidosis. To rule out high infection/sepsis. To rule evaluate ischemia or thromboembolic manifestations.	From 5 to 11 minutes [48]	Pre-hospital, hospital and anywhere.	In any acute case, fever, sepsis, infection.
POC influenza antigen (Ag)	To rule out influenza A and B.	10 to 15 minutes [49]	Pre-hospital, hospital and anywhere.	In fever and respiratory infection cases.
POC COVID Ag	To rule out COVID-19.	Few minutes	Pre-hospital, hospital and anywhere.	In any fever and respiratory infection case.
POC HIV	To rule out HIV.	About 20 minutes [50]	Pre-hospital, hospital and anywhere.	As needed in any sick case.
POC INR	To monitor international normalised ratio (INR) in patients who are on anticoagulant/warfarin therapy. To check the INR in any haemorrhage or case of bleeding. To check INR in the critical case before any medical or surgical intervention. [51]	Few minutes	Home care, pre-hospital and emergency departments.	Any time as the patient needs.
POC HbA1c	To monitor and reassess the diabetic patient. [52]	Few minutes	Home care, health centres, outpatient and emergency departments.	Any time as the patient needs.
POCP urine Trypsinogen	To diagnose pancreatitis in acute and emergency case as acute abdominal pain, vomiting and alcoholic patients. [31]	Few minutes	Emergency department.	Any time as patient need.
POC complete blood count (CBC)	To check haemoglobin (Hb) level. To check WBCs level. To check platelet level. To check haematocrit level. To check mean cell volume (MCV) - red cell. To check mean cell haemoglobin (MCH).	Few minutes	Emergency departments (ED), Acute care, intensive care unit (ICU) and outpatient department (OPD)	We can use it when we need rapid idea about the patient in ED, OPD or ICU. [53]
POC ultrasound (POCUS)	To detect pneumonia. To detect pneumothorax. To rule out plural effusion or pulmonary oedema. To evaluate the cardiac contractility. To rule out pericardial effusion. To rule out intra-abdominal free fluid. To rule out cholelithiasis. To rule out hydronephrosis or hydro ureter. To evaluate the ovary-uterine axis in female patients. To rule out urine retention. To rule out abdominal aortic aneurysm (AAA). To evaluate inferior vena cava (IVC) condition. To rule out deep venous thrombosis (DVT). [22]	Few minutes	Pre-hospital, hospital and anywhere.	It will be the future stethoscope, can use in any case.

The Advantages of POCT

**Figure 2:**
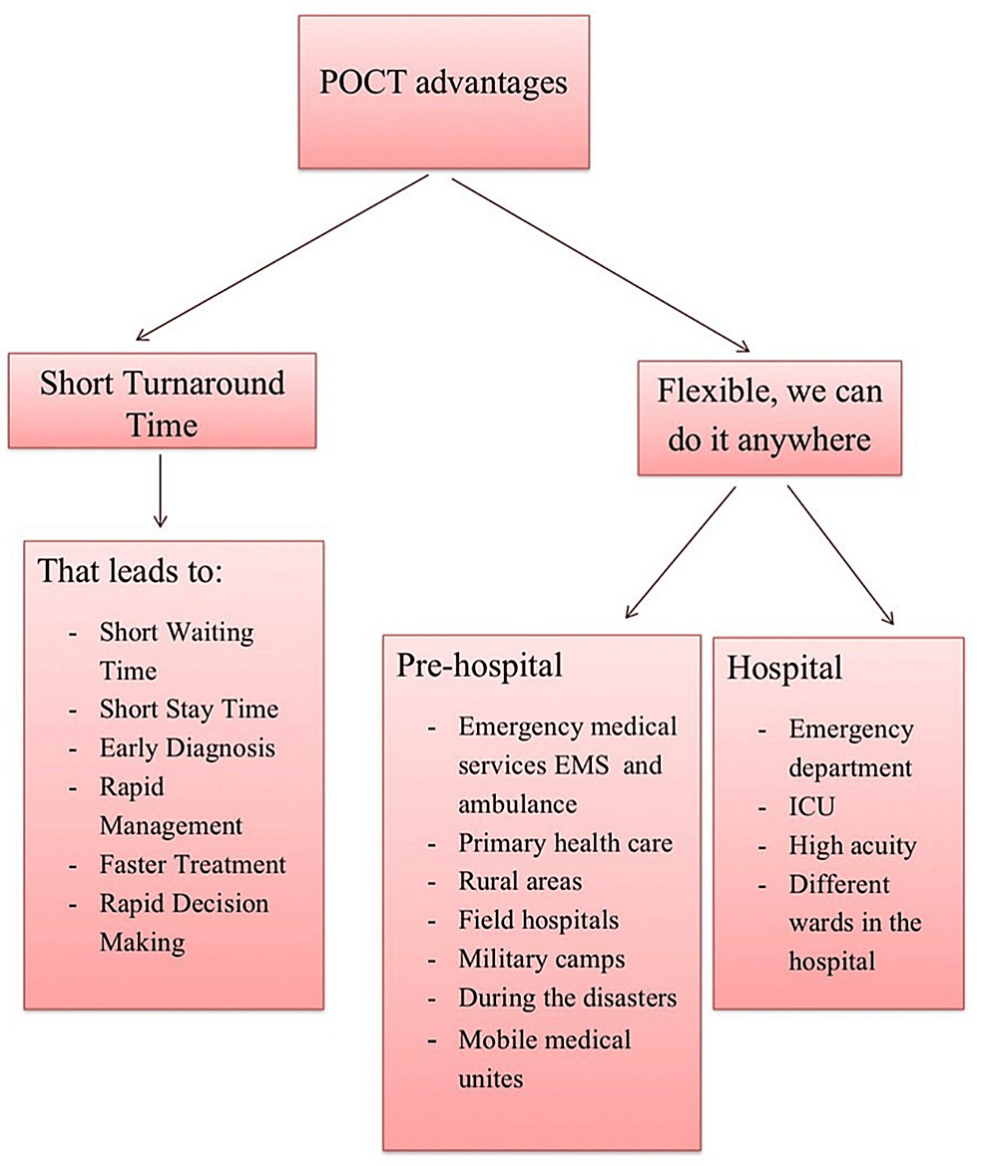
Advantages of POCT POCT: point-of-care testing, EMS: Emergency Medical Services, ICU: intensive care unit

The authors agreed that POCT has less turnaround time than the other tests sent to central laboratories or central departments, such as the clinical radiology department. This means that the time needed to get the result is significantly reduced with POCT. The flexibility of POCT is considered a notable advantage, as it can be performed virtually anywhere. However, there is a conflict regarding the cost-effectiveness of POCT. Some studies consider it as advantageous due to its low cost, while others consider it as a disadvantage due to its high cost. The rapid turnaround time of POCT offers several benefits, including reduced management time, quicker clinical decision-making, a rapid idea about critical patients, and enhanced efficiency in the triage process. Research suggests that the time patients stay in the ED decreases with the use of POCT. The flexibility of POCT has led to its use in pre-hospital settings, field hospitals, military or camp hospitals, and during disasters, mass gatherings and overcrowded areas (Figure 2) [13,14].

The Uses of POCT

Expanding on the advantages of POCT, it can be used anywhere in pre-hospital and hospital settings. In pre-hospital settings, POCT is utilized in ambulances, emergency medicine services, home care services, primary care services, field hospitals, military camps, casualty stations during disasters, clinical units in mass gatherings, and medical caravans in overcrowded areas. POCT can be used in EDs as frontline investigations. Additionally, it is employed in ICUs and various medical or surgical wards as needed [15-17].

Clinical Management With POCT

POCT is initiated, followed by an advanced test if necessary. POCT can be used as the initial test for sick or critical cases, providing a rapid initial diagnosis and enabling prompt, early, and proper management due to its shorter turnaround time. Mild and moderate cases requiring stabilizing and home treatment can depend solely on POCT for proper management. For severe cases needing further investigations or potential hospital admission, POCT can serve as the primary investigation for early management, followed by further investigations.

Clinical Cases That Can Be Managed Using POCT

Headache: If no red flags are present in the case of a headache, POCT can help to rule out differential diagnoses related to metabolic, infectious, and electrolyte imbalance causes of headaches. POC venous blood gases can provide insights into glucose levels, lactic acid levels, sodium, potassium, and even hemoglobin levels. POC blood count can rule out anemia, leukocytosis and thrombocytopenia. POC CRP is useful in ruling out inflammatory reactions. For headaches accompanied by tachycardia or irregular pulse detected by a pulse oximeter, an electrocardiogram is necessary. In case of headaches with red flags, POCT can offer a general understanding, but a computer tomography scan is required. Identification of red flags in headaches relies on the patient's history, clinical examinations, and the judgment of the healthcare professional [18,19].

Chest pain: In cases of chest pain, an ECG is required to rule out acute ischemic changes or abnormal cardiac rhythm. A portable chest X-ray is required to rule out lung opacity, pneumothorax, and pleural effusion. POC troponin T and I may be used to rule out acute coronary syndrome (ACS), and POC D-dimer may be used to rule out pulmonary embolism or aortic dissection. Both POC troponin and D-dimer have a shorter turnaround time than central laboratory testing. POC echocardiography is required to assess cardiac contractility, pericardial effusion, and aortic dissection. Abnormal contractility or hypokinesia in any cardiac segment may indicate an early sign of ACS. To rule out heart failure, POC proBNP and portable lung ultrasound are used to identify B-lines, indicating pleural effusion, or any hepatization, indicating pneumonia in the lung tissue. The presence of any red flag in clinical history, examination, and POCT findings may suggest further investigations and observation. Alternatively, ACS can be ruled out by POCT if the patient is young and has no risk factors, especially with atypical chest pain [20-22].

Upper respiratory tract infection (URTI): Some authors have emphasized the value of POC COVID-19 antigen and POC influenza virus antigens in the case of URTI without follicular tonsillitis. Patients with poor vital signs, including severe fever, tachycardia, or tachypnea, may need additional POCT, such as portable chest X-ray, neck soft tissue X-ray in cases of epiglottitis, POC venous blood gases, POC CRP, POC creatinine, POC complete blood count (CBC), and occasionally proBNP to rule out heart failure. POCT troponin T with ECG is required for any chest discomfort, even in cases of URTI. Some authors have suggested using POCUS in severe sore throat or neck pain to rule out any para-tonsillar collection. Further POCT or investigations may be required according to the clinical judgement and the severity of the case [23-26].

Lower respiratory tract infections (LRTI): In uncomplicated LRTI, POC COVID-19 antigen, influenza antigen, and chest X-ray may be sufficient for mild to moderate cases. However, in severe cases with high fever, tachycardia and tachypnea, additional POCT is required to evaluate these cases. These may include VBG, POC creatinine to rule out acute kidney injury (AKI) associated with severe infection, POC CBC, POC troponin to rule out pericarditis, ECG, POC CRP to determine the severity of inflammation, POC proBNP to rule out heart failure secondary to severe LRTI, and POCUS for heart and lung assessment [22-24,27-29].

High fever: In case of high fever, POCT can provide rapid insights into the patient’s condition, enabling the clinician to determine the need for further investigation. POC VBG is important for assessing lactic acid levels. POC CRP helps to assess the severity of inflammation. POC CBC evaluates white blood cells (WBCs), hemoglobin (Hb), and platelet levels. POC creatinine rules out AKI. POC troponin T rules out type II myocardial infarction, and POC proBNP may be required in cases of suspected heart failure. POCUS is helpful for checking the lung to rule out pleural effusion or pneumonia and assessing cardiac contractility and the pericardium. POCUS can also rule out abdominal free fluid, cholecystitis, cholelithiasis, and hydronephrosis. POCUS may be used to rule out fluid collection in cases of suspected abscesses. POC urine testing helps rule out urinary tract infections, and sometimes POC rapid malaria antigen testing is needed to exclude malaria. POC COVID-19 antigen and POC influenza tests can be used in cases of high fever [22,30].

Abdominal pain: Abdominal pain is a common emergency presentation that can be addressed using POCT to obtain a general idea about the patient. POCUS helps rule out free fluid, cholelithiasis or cholecystitis, abdominal aortic aneurysm (AAA), hydronephrosis, ovarian or uterine conditions, and to rule out urinary retention or cystitis. POC VBG is used for assessing lactic acid and electrolyte imbalance. POC creatinine helps to rule out AKI. POC CRP evaluates inflammatory reactions, and POC CBC assesses leukocytosis. POC urine trypsinogen helps to rule out pancreatitis, and POC troponin T helps rule out ACS. ECG may be required in cases of upper abdominal pain to rule out inferior MI. A portable chest X-ray is used to rule out pneumonia in upper abdominal pain or to detect air under the diaphragm in cases of perforated duodenal ulcer. A portable abdominal X-ray is used to rule out intestinal obstruction. POCT investigations should be selected according to the clinical judgment of the physician [22,31,30].

Shortness of breath (SOB): The stethoscope is used to assess wheezing. Subsequently, the pulse oximeter is used to determine oxygen saturation. An ECG is conducted to assess heart rate and cardiac rhythm and identify acute ischemic changes. POC echocardiography is used to rule out right ventricular dilatation as a result of pulmonary embolism and assess heart contractility to rule out heart failure or ACS. Additionally, POC troponin T and POC proBNP are used to rule out cardiac causes of SOB. POC VBG and POC D-dimer are used to rule out pulmonary embolism. A portable chest X-ray is used to identify lung opacity and pleural effusion. POCUS for the lungs to rule out pulmonary edema, pneumothorax and pleural effusion. POC creatinine is used to evaluate renal failure. POC CBC and CRP can be used to rule out infection. POC COVID-19 antigen and POC influenza tests can be used to rule out respiratory viral infections. The clinical judgement of the healthcare provider is crucial in determining the need for further investigations based on the patient’s condition [22,29,30].

Vomiting: Persistent vomiting can be investigated with POCT as follows: POC VBG to rule out acidosis or alkalosis and to check for electrolyte imbalance. POC RBG to rule out hypo- or hyperglycemia. POC ketones to rule out ketosis and POC creatinine to rule out AKI. POC CBC and CRP can be used to rule out infectious causes. POC troponin T can be used to rule out ACS. POC urine trypsinogen can be used to rule out pancreatitis. POCUS can be used in cases of upper abdominal pain or chest pain, as mentioned above (see Table 1). The attending clinician can determine if the patient needs further investigations or imaging based on the POCT results and the patient’s condition [22,30,31].

Back pain: Simple non-traumatic low back pain may not need investigation if the patient has no red flags in their history or clinical examination. However, back pain accompanied by red flags may need a portable X-ray, POC VBG, POC urine trypsinogen, POC CBC, POC CRP, POC erythrocyte sedimentation rate (ESR), POC creatinine, and occasionally POCUS [30-33].

Limb pain or swelling: In emergency cases of upper or lower limb pain or swelling, the underlying causes may include fractures, thromboembolic manifestations, or cellulitis. A portable X-ray is required to rule out fractures. POC D-dimer may be required to rule out deep venous thrombosis (DVT). POC CRP and CBC can be used to rule out cellulitis and infection as potential causes. POCUS can be used to rule out non-compressible veins in cases of DVT or to assess ischemia using Doppler ultrasound. POC VBG may be used to rule out lactic acidosis in severe infection or limb ischemia. Further investigations should be considered based on the results, the patient’s condition, and the clinical judgment of the attending physician [22,30].

Cellulitis: In cases of cellulitis, POC VBG, POC CRP, POC CBC, and POCUS are required for patient assessment [30].

Sepsis: Sepsis warrants a thorough investigation, and POCT can be helpful in the initial assessment of sepsis before starting the six-bundle management. POC VBG may be required to measure lactic acid levels, and POC CBC may be required to assess for leukocytosis and the patient’s hemoglobin. POC CRP can be used to assess the degree of infection. POC proBNP and POC troponin T can be used to evaluate the patient’s cardiovascular condition. An ECG is essential to assess cardiac rhythm, and POCUS can help to detect the site of infection (abdominal free fluid, cholecystitis, hydronephrosis, cystitis, pneumonia, pleural effusion, pericardial effusion, and any abscess) and evaluate the patient’s condition, such as assessing inferior vena cava (IVC) filling and cardiac contractility [22,30].

Acute critical illness: Acute critical cases can be assessed by POCT as follows: pulse oximeter to check oxygen saturation, ECG to assess cardiac rhythm, rate, and acute ischemic changes. POC VBG can be used to assess lactic acid, electrolytes, pH, and sodium bicarbonate. POC CRP can be used to evaluate infection causes. POC CBC can be used to assess leukocyte, hemoglobin and platelet levels. POC D-dimer can be used to rule out pulmonary embolism, aortic dissection, or thromboembolic manifestations. POC troponin T can be used to rule out ACS. POC proBNP can be used to rule out heart failure. A portable chest X-ray is useful to rule out lung opacities, pleural effusion, or pneumothorax. POCUS is utilized to check heart contractility, precordium, and right ventricle. POCUS is employed to assess lung conditions, such as pulmonary edema, effusion, pneumothorax, and pneumonia. POCUS is also used to assess the IVC, AAA, abdominal free fluid, hydronephrosis, and DVT. POC ketones can be used to rule out ketosis [22,30].

Osteomyelitis, arthritis, and septic arthritis: These can be assessed using POC VBG, CBC, CRP, and POCUS. A portable X-ray is important in detecting osteomyelitis and osteoarthritis.

Disadvantages of POCT

**Figure 3:**
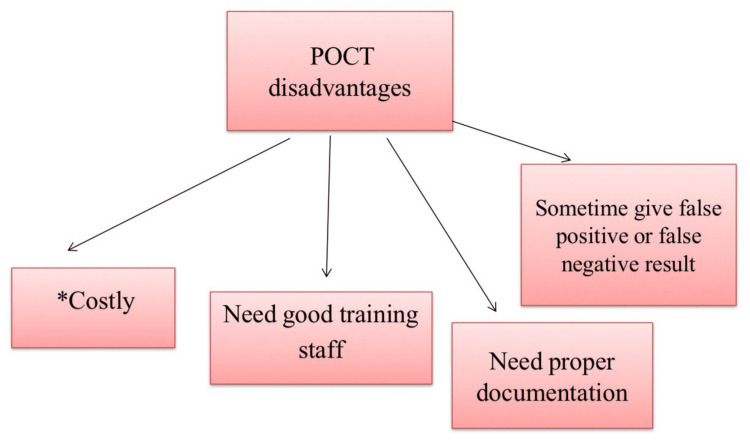
Disadvantages of point-of-care testing (POCT) *Some studies have mentioned that POCT is reducing the cost of emergency services but other studies have denied this [3,6,11,34].

Some studies have mentioned the disadvantages of POCT, such as the need for training those who will use POCT, high costs, difficulties in documentation, and occasional inaccurate results, and other studies have found no difference in time of stay in the ED with POCT based on their experience (Figure 3). For explanation, some authors mention the POCT could be costly and others denied this. Depending on some sequence of events POCT may be costly. For example, if one patient presented for any medical complaint and he or she did the POCTs, if it was a positive finding that led to further investigations in the central lab, it means he should pay the cost of the POCT and the central lab test. Maybe he can pay less if he pays the cost of the central lab test only. On the other hand, if one patient presented for a medical complaint, then he or she did the POCTs, and the doctor's decision was home treatment with no need for further investigations, which means the patient will pay the fee of the POCT only, which is less than the fee of the central lab test. The POCT instrument, reagents, and maintenance should be considered part of the cost. POCT has difficulties with documentation, such as no connection between the POCT machines and the patient's electronic record, which leads to manual documentation. Manual documentations sometimes get mistaken in the record as wrong value recorded or recorded in another patient's file [3,6,11,34-36].

How to Get High-Quality Results From POCT Without Clinical Errors

**Figure 4:**
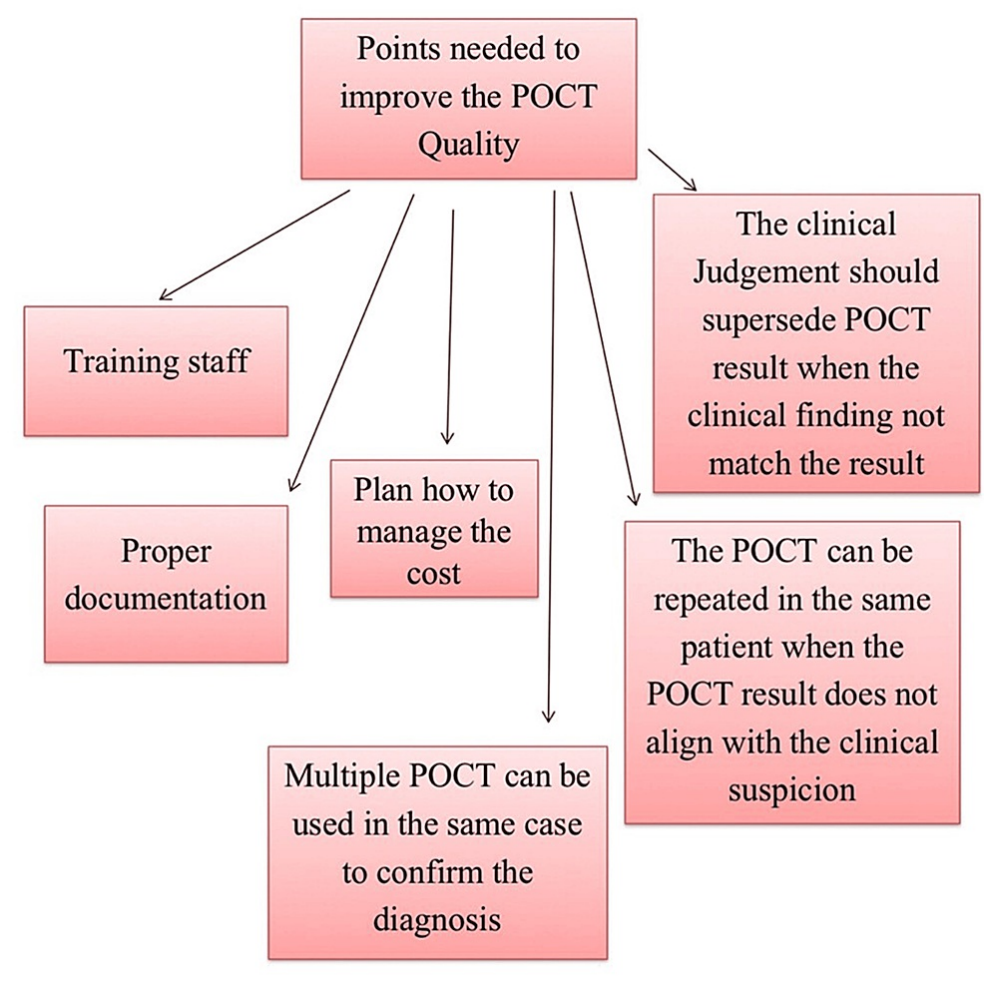
Points to improve point-of-care testing (POCT) quality

Considering the advantages and disadvantages of POCT, we can make policies on how to use it. Staff require training to use POCT, and proper documentation is essential. The cost is controversial; some studies mention low costs, while others report high costs. Clinical managers or administrators should plan how to use it, considering the cost. POCT occasionally gives false-positive results. Therefore, the physician’s clinical judgment, the patient’s condition, and POCT investigations should be integrated to reach a clinical decision. Multiple POCT can be used in the same case to confirm the diagnosis, or the POCT can be repeated in the same patient when the POCT result does not align with the clinical suspicion. Clinical judgment should supersede POCT results in determining whether the patient needs further investigations (Figure 4) [34-40]. 

Discussion

The crucial aspect of the POCT subject is the time factor. POCT has a shorter turnaround time than conventional laboratory test, meaning that the time needed to obtain results is significantly reduced, often taking only a few minutes. In healthcare services, time is of utmost importance when managing medical cases in the ED, especially in overcrowded areas. Prolonged waiting time in the ED can contribute to overcrowding, leading to numerous complications, such as insufficient time for thorough examinations in some cases, delays in treatment, and delays in minor surgical procedures. Overcrowded ED may also experience delays in subspecialty consultations, as well as delays in patient disposition and medical decision-making. In healthcare service, the goal is to minimize unnecessary waiting times, facilitate early management, and prevent any delays in healthcare delivery. This approach aims to prevent the complications associated with overcrowding, optimize patient management, and address each case with the appropriate priority within the required timeframe. Therefore, when we receive test results quickly, we can initiate early management and provide rapid treatment for critically ill cases. Early discharge with proper management becomes feasible, allowing us to handle more patients in the ED. POCT proves highly helpful in overcrowded hospitals and medical centers. The short turnaround time assists medical decision-makers in efficient triage decisions in the ED. Additionally, it facilitates early diagnosis, enabling faster initiation of supportive management for patients [14,20,41].

In triage, POCT can direct the case along the path of proper management without unnecessary investigations. For example, in a case of high fever with tachycardia, conducting COVID-19 and influenza antigen tests can help to diagnose respiratory viral infection. A positive result may indicate the need for supportive treatment without further investigations. Another case of high fever, high POC lactic acid and CRP suggests we initiate the six-bundle management for sepsis early. In some medical centers, POC troponin is employed in triage for patients with chest pain. A positive result prompts the transfer of the case to the cardiac unit, while a negative result may lead to transferring the patient to the observation unit. At the frontline of the ED, we can gain insights into managing specific cases. For example, in a patient with leg swelling, we may rule out cellulitis or DVT using POC D-dimer, CRP and CBS. Patients experiencing symptoms such as vomiting, abdominal pain, chest pain or flank pain can benefit from reduced waiting times through POCT, facilitating a quicker diagnosis and enabling early management with timely hospital departure or disposition. For example, POCUS can be used to identify conditions such as hydronephrosis, cholelithiasis, AAA, abdominal free fluid, pericardial effusion and pneumonia. POC creatinine can be used to assess for AKI. In cases of acute abdomen, POC CBC and CRP can aid in diagnosing severe infections, ruling out appendicitis. POC troponin is used to rule out ACS, and POC D-dimer is often used to rule out aortic dissection or pulmonary embolism. Decision-makers can use POC creatinine to determine the need for contrast-enhanced CT in some patients. Additionally, POC INR and PTT can be helpful for patients who are on warfarin therapy or for stroke patients needing early thrombolytic therapy [4,7,9,15-17,21,24].

Another crucial aspect of POCT is its flexibility; it can be performed anywhere, at any time, and in close proximity to the patient. Some studies in emergency medical services (EMS) advised the use of POCT troponin to determine which patients can be transferred to a heart hospital or a general hospital. Home care services can use POCT for home care and bedridden patients, assessing electrolytes, pH, ketones, CRP, creatinine, and various POCTs to evaluate the patient’s condition at home. During mass gatherings, POCT can be employed in medical mobile units to investigate and manage many individuals, reducing the number of patients transferred to hospitals. During disasters, POCT can be used to triage, investigate and manage causalities, reducing the number of patients transferred to hospitals. In the ED, POCT can be used as a frontline to triage and investigate ambulatory cases. In the ICU, resuscitation room, and high acuity unit, physicians can gain rapid insights into critical cases with POCT’s short turnaround time. For example, in the case of SOB, we can determine pH, D-dimer, proBNP, troponin, blood gases, X-ray, and ultrasound using POCT, allowing us to identify the cause of SOB, whether it be ACS, pulmonary embolism, heart failure, acidosis, or chest infection, and start supportive treatment as early as possible [1,5,6,8,12,15,16,30,42].

POCUS offers multiple benefits when used by well-trained physicians. The pocket-sized ultrasound is regarded as the future stethoscope. With POCUS, we can examine the chest to rule out pneumothorax, pulmonary edema, and pneumonia. POCUS is also valuable for assessing the heart to rule out pericardial effusion, abnormal contractility, or abnormal heart motion that may indicate cardiac ischemia, a dilated right ventricle in cases of suspected pulmonary embolism, and aortic dissection. In the abdomen, POCUS can be employed to rule out cholelithiasis, cholecystitis, AAA, hydronephrosis, and urinary retention, evaluate the caliber of the IVC, and identify abdominal free fluid. Additionally, we can evaluate the ovaries and uterus using POCUS. DVT can also be detected using POCUS [12,22,29].

We can designate POCT in our ED as frontline management for all ambulatory patients. This helps to triage patients, conduct early and rapid investigations, decrease the turnaround time and deliver prompt treatment. We aim to efficiently assess and treat more ambulatory patients, ensuring a short stay time without unnecessary delays or prolonged waiting periods [2,6,7].

When using POCT for diagnosis, we can use multiple POCTs to confirm our diagnostic findings. For example, a diagnosis of heart failure can be made using POCT proBNP and POCUS. Ischemic heart disease can be diagnosed using ECG, POCUS, and POC troponin. In cases of pneumonia or lower respiratory tract infections, we can use POC blood gases, POCUS, POC proBNP, portable chest X-ray, POC CRP, POC CBC, and POC respiratory virus antigens to make a diagnosis. To confirm a diagnosis of DVT, a combination of POCUS and POC D-dimer can be used. In the case of pulmonary embolism, a probable diagnosis can be made using ECG, POCUS, VBG, and POC D-dimer. This allows for the initiation of anticoagulant therapy before transferring the patient for a CT angiography to confirm the diagnosis at a tertiary hospital [4-8,12,29,30].

To ensure high-quality POCT results, it is essential to have trained staff operating the POC machines. Accurate documentation is important to review the results during decision-making, ensuring results are not assigned to the wrong patient. Some medical centers consider POCT as a cost-effective option for daily services, while others perceive it as a more expensive option. The cost of POCT needs careful evaluation and consideration based on the specific medical center’s circumstances. For sound medical decisions, the use of multiple POCTs may be necessary to confirm medical diagnoses. We can repeat the POCT if the initial result does not align with clinical findings. Additionally, clinical findings and judgment take precedence over POCT results; if there is a discrepancy or if they indicate the need for further investigations, they should guide the decision-making process [4,9,11,13,34,35,37,38].

Study recommendation

We recommend POCT as the frontline investigation in EDs, primary healthcare, rural areas, military camps, field hospitals, and pre-hospitals. It may help in triage in the pre-hospital setting with less patient transferal to tertiary hospitals. This approach can help in triaging patients in EDs and fast-track areas. It also helps to decrease the waiting time by enabling the examination of more patients in fast-track areas in EDs. Ultimately, POCT can decrease the time of stay in EDs, facilitating early diagnosis, management, treatment, and deposition.

## Conclusions

With careful consideration, we can leverage POCT to improve healthcare services. Its short turnaround time and flexibility enable its use in various settings. The quick turnaround time leads to rapid availability of results, contributing to shorter stays and reduced waiting time that lead to more patient management - particularly beneficial in overcrowded areas. POCT provides an early understanding of critical cases, allowing for prompt initiation of appropriate treatment while awaiting the results of advanced investigations.

## References

[1] Florkowski C, Don-Wauchope A, Gimenez N, Rodriguez-Capote K, Wils J, Zemlin A (2017). Point-of-care testing (POCT) and evidence-based laboratory medicine (EBLM) - does it leverage any advantage in clinical decision making?. Crit Rev Clin Lab Sci.

[2] Kost GJ (1995). Guidelines for point-of-care testing. Improving patient outcomes. Am J Clin Pathol.

[3] Junker R, Schlebusch H, Luppa PB (2010). Point-of-care testing in hospitals and primary care. Dtsch Arztebl Int.

[4] Isbell TS, Colwell E, Frank EL, Karon BS, Luzzi V, Wyer LA (2021). Professional certification in point-of-care testing. EJIFCC.

[5] Fermann GJ, Suyama J (2002). Point of care testing in the emergency department. J Emerg Med.

[6] St John, Andrew Andrew, and Christopher P. Price (2018). Benefits of point-of-care testing in the Emergency Department. https://acutecaretesting.org/en/articles/benefits-of-point-of-care-testing-in-the-emergency-department.

[7] Singer AJ, Taylor M, LeBlanc D, Meyers K, Perez K, Thode HC Jr, Pines JM (2018). Early point-of-care testing at triage reduces care time in stable adult emergency department patients. J Emerg Med.

[8] Castro HJ, Oropello JM, Halpern N (1995). Point-of-care testing in the intensive care unit. The intensive care physician's perspective. Am J Clin Pathol.

[9] Shephard MD, Mazzachi BC, Watkinson L (2009). Evaluation of a training program for device operators in the Australian Government's Point of Care Testing in General Practice Trial: issues and implications for rural and remote practices. Rural Remote Health.

[10] Díaz-Garzón J, Oliver P, Crespo G (2020). Experience on how to implement a preanalytical and POCT unit in Madrid's IFEMA field hospital during this unprecedented COVID-19 emergency. Biochem Med (Zagreb).

[11] Ehrmeyer SS (2011). Plan for quality to improve patient safety at the point of care. Ann Saudi Med.

[12] Weinberg I, Olin J, Jaff MR (2022). Point-of-care ultrasonography. N Engl J Med.

[13] Parvin CA, Lo SF, Deuser SM, Weaver LG, Lewis LM, Scott MG (1996). Impact of point-of-care testing on patients' length of stay in a large emergency department. Clin Chem.

[14] Rooney KD, Schilling UM (2014). Point-of-care testing in the overcrowded emergency department--can it make a difference?. Crit Care.

[15] Sohn AJ, Hickner JM, Alem F (2016). Use of point-of-care tests (POCTs) by US primary care physicians. J Am Board Fam Med.

[16] Hicks JM, Haeckel R, Price CP, Lewandrowski K, Wu AH (2001). Recommendations and opinions for the use of point-of-care testing for hospitals and primary care: summary of a 1999 symposium. Clin Chim Acta.

[17] Park HD (2021). Current status of clinical application of point-of-care testing. Arch Pathol Lab Med.

[18] Minen MT, Robbins MS, Loder E (2020). Addressing the crisis of diagnosis and management of migraine in primary care: a summary of the American Headache Society FrontLine Primary Care Advisory Board. Headache.

[19] Giamberardino MA, Affaitati G, Costantini R, Guglielmetti M, Martelletti P (2020). Acute headache management in emergency department. A narrative review. Intern Emerg Med.

[20] Singer AJ, Ardise J, Gulla J, Cangro J (2005). Point-of-care testing reduces length of stay in emergency department chest pain patients. Ann Emerg Med.

[21] Nilsson S, Andersson PO, Borgquist L (2013). Point-of-care troponin T testing in the management of patients with chest pain in the Swedish primary care. Int J Family Med.

[22] Hashim A, Tahir MJ, Ullah I, Asghar MS, Siddiqi H, Yousaf Z (2021). The utility of point of care ultrasonography (POCUS). Ann Med Surg (Lond).

[23] Brigadoi G, Gastaldi A, Moi M (2022). Point-of-care and rapid tests for the etiological diagnosis of respiratory tract infections in children: a systematic review and meta-analysis. Antibiotics (Basel).

[24] Cooke J, Llor C, Hopstaken R, Dryden M, Butler C (2020). Respiratory tract infections (RTIs) in primary care: narrative review of C reactive protein (CRP) point-of-care testing (POCT) and antibacterial use in patients who present with symptoms of RTI. BMJ Open Respir Res.

[25] Song Q, Sun X, Dai Z (2021). Point-of-care testing detection methods for COVID-19. Lab Chip.

[26] Furukawa M, Hashimoto K, Kitani Y, Yoshida M (2022). Point-of-care ultrasound in the head and neck region. J Med Ultrason (2001).

[27] Wood F, Brookes-Howell L, Hood K (2011). A multi-country qualitative study of clinicians' and patients' views on point of care tests for lower respiratory tract infection. Fam Pract.

[28] Cooke J, Butler C, Hopstaken R (2015). Narrative review of primary care point-of-care testing (POCT) and antibacterial use in respiratory tract infection (RTI). BMJ Open Respir Res.

[29] Gartlehner G, Wagner G, Affengruber L (2021). Point-of-care ultrasonography in patients with acute dyspnea: an evidence report for a clinical practice guideline by the American College of Physicians. Ann Intern Med.

[30] Damhorst GL, Tyburski EA, Brand O, Martin GS, Lam WA (2019). Diagnosis of acute serious illness: the role of point-of-care technologies. Curr Opin Biomed Eng.

[31] Jang T, Uzbielo A, Sineff S, Naunheim R, Scott MG, Lewis LM (2007). Point-of-care urine trypsinogen testing for the diagnosis of pancreatitis. Acad Emerg Med.

[32] Islam E, Hani A, Muayad K (2020). Non-traumatic low back pain: five case reports and Al Wakra recommendations. Arch Clin Med Case Rep.

[33] Casser HR, Seddigh S, Rauschmann M (2016). Acute lumbar back pain. Dtsch Arztebl Int.

[34] Guarner J, Jenkins KM, Franks NM (2018). Successful and unsuccessful point-of-care testing in the emergency room. Am J Clin Pathol.

[35] Gentilotti E, De Nardo P, Cremonini E (2022). Diagnostic accuracy of point-of-care tests in acute community-acquired lower respiratory tract infections. A systematic review and meta-analysis. Clin Microbiol Infect.

[36] Alter DN (2021). Point-of-care testing for the emergency department patient: quantity and quality of the available evidence. Arch Pathol Lab Med.

[37] Price CP, Smith I, Van den Bruel A (2018). Improving the quality of point-of-care testing. Fam Pract.

[38] Liikanen E, Lehto L (2013). Training of nurses in point-of-care testing: a systematic review of the literature. J Clin Nurs.

[39] Wiencek J, Nichols J (2016). Issues in the practical implementation of POCT: overcoming challenges. Expert Rev Mol Diagn.

[40] McIntosh BW, Vasek J, Taylor M, Le Blanc D, Thode HC, Singer AJ (2018). Accuracy of bedside point of care testing in critical emergency department patients. Am J Emerg Med.

[41] Elrobaa IH, Dafalla EH, Khalid MK, Kutty MF (2021). Al Wakra type II myocardial infarction-a case report in our emergency department. AME Case Rep.

[42] Camaro C, Aarts GW, Adang EM (2023). Rule-out of non-ST-segment elevation acute coronary syndrome by a single, pre-hospital troponin measurement: a randomized trial. Eur Heart J.

[43] Jain A, Subhan I, Joshi M (2009). Comparison of the point-of-care blood gas analyzer versus the laboratory auto-analyzer for the measurement of electrolytes. Int J Emerg Med.

[44] Bugge C, Sether EM, Pahle A, Halvorsen S, Sonbo Kristiansen I (2018). Diagnosing heart failure with NT-proBNP point-of-care testing: lower costs and better outcomes. A decision analytic study. BJGP Open.

[45] Cullen L, Collinson PO, Giannitsis E (2022). Point-of-care testing with high-sensitivity cardiac troponin assays: the challenges and opportunities. Emerg Med J.

[46] (2023). D-dimer: the biomarker of choice to aid the diagnosis of venous thromboembolism. https://www.radiometer.com/en/products/immunoassay-testing/aqt90-flex-immunoassay-analyzer/d-dimer-test-on-the-aqt90-flex-immunoassay-analyzer.

[47] Vilaine E, Gabarre P, Beauchet A (2021). Point-of-care capillary blood creatinine: a prospective study in cardiology and nephrology outpatients. Cardiol Cardiovasc Med.

[48] Ismail F, Mackay WG, Kerry A, Staines H, Rooney KD (2015). The accuracy and timeliness of a point of care lactate measurement in patients with sepsis. Scand J Trauma Resusc Emerg Med.

[49] Azar MM, Landry ML (2018). Detection of influenza A and B viruses and respiratory syncytial virus by use of Clinical Laboratory Improvement Amendments of 1988 (CLIA)-waived point-of-care assays: a paradigm shift to molecular tests. J Clin Microbiol.

[50] Arora DR, Maheshwari M, Arora B (2013). Rapid point-of-care testing for detection of HIV and clinical monitoring. ISRN AIDS.

[51] (2009). Point-of-care international normalized ratio (INR) monitoring devices for patients on long-term oral anticoagulation therapy: an evidence-based analysis. Ont Health Technol Assess Ser.

[52] Schnell O, Crocker JB, Weng J (2017). Impact of HbA1c testing at point of care on diabetes management. J Diabetes Sci Technol.

[53] Rao LV, Ekberg BA, Connor D, Jakubiak F, Vallaro GM, Snyder M (2008). Evaluation of a new point of care automated complete blood count (CBC) analyzer in various clinical settings. Clin Chim Acta.

